# Programmable multi-mode entanglement via dissipative engineering in vibrating trapped ions

**DOI:** 10.1126/sciadv.adv7838

**Published:** 2025-07-02

**Authors:** Yue Li, Yi Li, Xu Cheng, Lingna Wang, Xingyu Zhao, Waner Hou, Kamran Rehan, Mingdong Zhu, Lin Yan, Xi Qin, Xinhua Peng, Haidong Yuan, Yiheng Lin, Jiangfeng Du

**Affiliations:** ^1^ CAS Key Laboratory of Microscale Magnetic Resonance and School of Physical Sciences, University of Science and Technology of China, Hefei 230026, China.; ^2^ Anhui Province Key Laboratory of Scientific Instrument Development and Application, University of Science and Technology of China, Hefei 230026, China.; ^3^ Hefei National Laboratory, University of Science and Technology of China, Hefei 230088, China.; ^4^ Department of Mechanical and Automation Engineering, The Chinese University of Hong Kong, Shatin, Hong Kong.; ^5^ Institute of Quantum Sensing and School of Physics, Zhejiang University, Hangzhou 310027, China.

## Abstract

Generating multi-partite quantum entangled states amid dissipative environment is essential for advancing quantum simulation, metrology, and fundamental quantum research. Here, we use controlled dissipation as a resource and present an approach to generate programmable multimode entangled states in the vibrational modes of trapped ions, free of stringent requirements of initial state preparation. We experimentally demonstrate the generation of multimode squeezed states across two, three, and five modes out of initial thermal states by controlled couplings to dissipative internal spins. We characterize the output states by fidelity estimates and two-body correlations, confirming genuine multipartite entanglement using the van Loock-Furusawa inseparability criteria. This work outlines a generic path to create entangled nonclassical states with quantum harmonic oscillators, applicable for quantum information processing in continuous-variable quantum systems.

## Teaser

Engineered dissipation is applied to vibrating trapped ions, stably generating entanglement across multiple motional modes.

## INTRODUCTION

Multipartite entanglement (*1*) is a key resource for the research on fundamental quantum mechanics (*2*) and quantum-enhanced information processing. It has a wide range of applications in quantum computation (*3*), communication (*4*), simulation (*5*), and sensing (*6*). Recent advances include production of scaled cluster states (*7*), Greenberger-Horne-Zeilinger states (*8*), collective spin-squeezed state with two-axis twisting (*9*), large-scale twin-fock state (*10*) via quantum phase transition, non-Gaussian states (*11*), and graph states (*12*) by heralding on detection incidence. While substantial efforts have been made to isolate the system from the dissipative environment, an alternative and potentially more robust approach is instead embracing the dissipation as a resource, known as reservoir engineering or dissipative engineering (*13*–*15*). By introducing a controlled dissipative bath with tailored bath-system couplings, the system could be pumped to the desired state. In various systems with multiple quantum bits (qubits), recent theoretical and experimental advances show promising applications in entangled state production and quantum simulation (*16*–*25*). In continuous variable systems, on the other hand, pioneering theoretical (*26*) and experimental works (*27*–*30*) lay the foundation for single-mode and two-mode dissipative engineering. Moreover, several theoretical proposals have been put forward for dissipative entanglement generation in multimode bosonic systems (*31*–*33*); however, this remains experimentally challenging. In addition, given the possibility of exploring many interesting states in larger systems for a range of applications, a programmable method while harnessing the coherent and dissipative operations would provide a useful toolbox.

Here, we present a programmable and deterministic dissipative generation of bosonic Gaussian states using vibrating trapped ions, and we experimentally demonstrate the production of multimode entanglement. We stroboscopically apply programmed combinations of coherent couplings between the motional modes and the mutual spins, interleaved by dissipation on the spin. This technique belongs to the general category of Trotterization in open quantum system (*34*). In our demonstration, the overall process effectively generates a series of dissipative pumping on the ion motion, described by a set of commuting multimode Lindblad operators. Despite the large dimensional Hilbert space, the resulting pumping process creates a flow of populations from all states toward a sole dark state, which is the desired entangled state. The number of pumps is same with the number of modes; thus, our method could be readily scaled. In the experiment, we start from initial thermal states and focus on creating N*-*mode entangled states resembling the multimode squeezed state (*35*), which is also the cluster state in the continuous-variable regime (*36*, *37*), an essential building block for measurement-based quantum computation. We produce entanglement of this state with N=2,3,5 in the radial modes of the ion chain and analyze the state fidelity and the phonon correlation, respectively. The genuine-multipartite-entanglement of these states is verified through the van Loock-Furusawa inseparability criteria (*38*–*40*). Our method can be applied to more ions with more motional modes and may be applied to other experimental platforms with interactions between bosonic modes and dissipative qubits, such as superconducting cavities (*41*), atomic ensembles (*30*, *42*), and nanomechanics (*28*).

**Fig. 1.**
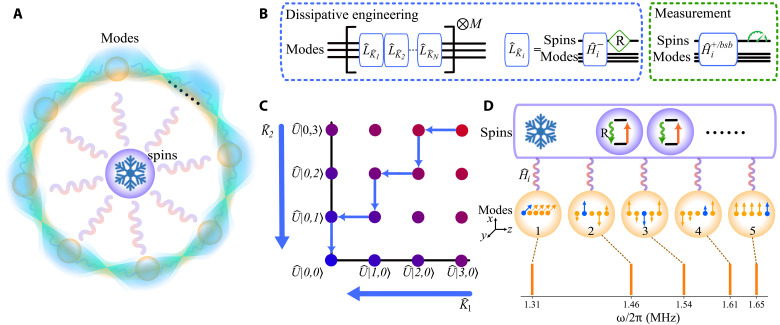
Programmable multimode entanglement generation by dissipative engineering. (**A**) Dissipative mutual spins coupled to multiple modes, generating entanglement between the modes. (**B**) Procedure for the dissipative engineering, constructed by the Trotterization process with each effective dissipative step denoted as ${\hat{L}}_{{\hat{K}}_{i}}$, with M repetitions. Each dissipative step is constructed by coherent spin motion coupling ${\hat{H}}_{i}^{-}$, followed by the spin reset–labeled “R.” Typical measurements of the modes are achieved by applying another set of spin-motion couplings ${\hat{H}}_{i}^{+}$ or ${\hat{H}}_{i}^{\text{bsb}}$ in the text, followed by spin readout. (**C**) An example of a two-mode pumping process. Sequential application of effective quantum jump ${\hat{K}}_{1,2}$ dissipatively drives the motion along the transformed lattice space, eventually reaching the desired entangled ground state $|\;\psi \;\rangle =\hat{U}|\;0,0\;\rangle$. (**D**) Illustration of radial mode structure and spin couplings. The spectrum shows frequency-resolved motional modes for the case of five trapped ions. The motion-spin couplings in (A) could be programmed by applying combinations of multiple laser fields resonant with each mode. These couplings drive the motional modes collectively and transfer unwanted excitation to the spins, labeled as the orange arrows. Thus, optical pumping on the spin, denoted as R and green squiggle arrow, creates one of the effective jump operators ${\hat{K}}_{i}$ in the text. Here, we use the radial modes vibrating along *x* and *y* directions. The arrows indicate the mode vectors and the length represents the strength of spin motion couplings. To detect the phonon correlation, we map each mode’s population into the corresponding spins, labeled in blue, for state readout with site-resolved detection.

We consider N bosonic modes with the annihilation and creation operators ${\left\{{\hat{a}}_{i},{\hat{a}}_{i}^{†}\right\}}_{i=1}^{N}$, as the elements of 1×N vectorial operators $\hat{a}\equiv \left({\hat{a}}_{1},{\hat{a}}_{2},\ldots ,{\hat{a}}_{N}\right)$ and ${\hat{a}}^{†}\equiv \left({\hat{a}}_{1}^{†},{\hat{a}}_{2}^{†},\ldots ,{\hat{a}}_{N}^{†}\right)$, with the vacuum ground state denoted as $\left|0^{⊗N}\rangle \right.$. Here, we extend the transformation method of a single mode (*13*, *27*) to multiple modes through Bogoliubov transformation. Particularly, we focus on a transformation $\hat{U}=\text{exp}\left[\frac{r}{2}\sum_{i,j}G_{i,j}\left({\hat{a}}_{i}{\hat{a}}_{j}-{\hat{a}}_{i}^{†}{\hat{a}}_{j}^{†}\right)\right]$, where the real number r denotes the squeezing parameter and G is a symmetric matrix. Thus, one can obtain the Gaussian annihilation operators $\hat{K}=\left({\hat{K}}_{1},{\hat{K}}_{2},\ldots ,{\hat{K}}_{N}\right)$ from $\hat{a}$, denoted as

$$\begin{array}{c} \hat{a}\to \hat{K}\equiv \hat{U}\hat{a}{\hat{U}}^{†}=\hat{a}A+{\hat{a}}^{†}B,\\ |\;0^{⊗N}\rangle \to |\;\psi \;\rangle \equiv \hat{U}|\;0^{⊗N}\;\rangle \end{array}$$ (1)

where A and B are N×N complex matrices and ∣ψ〉 is the desired transformed ground state with respect to unitary $\hat{U}$. A series of applications of ${\hat{a}}_{i}$ would cool the system to the ground state since ${\hat{a}}_{i}\left|\;,0^{⊗N}\rangle \right.=0$. Similarly, if one creates a set of pumping containing all N operators ${\hat{K}}_{i}$, then the desired ∣ψ〉 would be produced as the only dark state since ${\hat{K}}_{i}|\psi \rangle =0$, as seen in Eq. 1. To form effective dynamics where multiple jump operators are simultaneously applied, in general, the Trotterization approach could be used (*34*), so that the desired operation $e^{\sum_{i}{\hat{L}}_{{\hat{K}}_{i}}t}$ could be approximated by ${\left(\prod_{i}e^{{\hat{L}}_{{\hat{K}}_{i}}t/M}\right)}^{M}$, where the incoherent Liouvillian ${\hat{L}}_{{\hat{K}}_{i}}\left[\hat{\rho }\right]={\hat{L}}_{{\hat{K}}_{i}}\hat{\rho }{\hat{L}}_{{\hat{K}}_{i}}^{†}-\frac{1}{2}\left\{{\hat{L}}_{{\hat{K}}_{i}}^{†}{\hat{L}}_{{\hat{K}}_{i}},\hat{\rho }\right\}$ describes the dissipative operation applied to density operator $\hat{\rho }$. A duration of t/M for each jump is applied sequentially and with overall M repetitions, and ${\hat{L}}_{{\hat{K}}_{i}}=\sqrt{\gamma_{i}}{\hat{K}}_{i}$ with strength $\gamma_{i}$. Each jump operation can be achieved by introducing the ancilla spins as the environment, as depicted in Fig. 1A, implemented through generating the system-environment unitary evolution $e^{-i{\hat{H}}_{i}^{-}t/M}$ followed by resetting the ancilla spins, with

$${\hat{H}}_{i}^{-}=\frac{\Omega_{i,j}^{-}}{2}{\hat{\sigma }}_{j}^{+}{\hat{K}}_{i}+h.c.$$ (2)

where $\Omega_{i,j}^{-}$ is the Rabi frequency, ${\hat{\sigma }}_{j}^{\pm }$ is the *j*th spin flip operators, h.c. denotes Hermitian conjugates, and we set ћ=1. By applying both ${\hat{H}}_{i}^{-}$ and dissipation on the spin denoted with quantum jump operator ${\hat{\sigma }}_{j}^{-}$, with proper parameters, an effective jump operator could be created proportional to ${\hat{K}}_{i}$ by adiabatic elimination of the spin (*14*), thus implementing ${\hat{L}}_{{\hat{K}}_{i}}$. Here, we apply the coherent drive and dissipation stroboscopically, as shown in Fig. 1B, which would give a dynamics resembling the effective jumps ${\hat{K}}_{i}$ sequentially. Such a sequence would pump the motional state to the desired state ∣ψ〉, as illustrated in Fig. 1C for the case of two modes.

## RESULTS

For a demonstration, we generate N-mode squeezed state with all-to-all connectivity in the trapped-ion system, a type of cluster state in the continuous-variable regime. These states can be represented by $|\;\psi \;\rangle \equiv \hat{U}|\;0^{⊗N}\;\rangle$ in Eq. 1, here with $\hat{U}=\text{exp}\left[\frac{r}{2}\sum_{i,j}G_{i,j}\left({\hat{a}}_{i}{\hat{a}}_{j}-{\hat{a}}_{i}^{†}{\hat{a}}_{j}^{†}\right)\right]$, where G is the adjacency symmetric matrix with elements of either 1 or 0 that encodes the joint squeezing between two of the modes. In this case, we set all Rabi rates equal and denote their value as $\Omega^{-}$, and the modified Hamiltonian and the transformed annihilation operator are then written as (see the Supplementary Materials for details)

$$\begin{array}{c} {\hat{H}}_{i}^{-}=\frac{\Omega^{-}}{2}{\hat{\sigma }}_{j}^{+}{\hat{K}}_{i}+h.c.,\\ {\hat{K}}_{i}=\text{cosh}\left(r\right)\;{\hat{a}}_{i}+\left\{\frac{\text{cosh}\left[\left(N-1\right)r\right]-\text{cosh}\left(r\right)}{N}\right\}\sum_{k=1}^{N}{\hat{a}}_{k}\\ -\text{sinh}\left(r\right)\;{\hat{a}}_{i}^{†}+\left\{\frac{\text{sinh}\left[\left(N-1\right)r\right]+\text{sinh}\left(r\right)}{N}\right\}\sum_{k=1}^{N}{\hat{a}}_{k}^{†} \end{array}$$ (3)

Thus, a coherent drive combining Eq. 2 and Eq. 3 upon dissipation mentioned above gives effective pumps in the form of ${\hat{K}}_{i}$. Successive application of these pumps according to Trotterization then leads to the desired N-mode squeezed state.

Experimentally, we use trapped ^40^Ca^+^ ions with the collective motional modes as the system and internal spins as the reservoir. The internal spins are denoted as $\;|\;↓\;\rangle \equiv |\;L=0,J=1/2,M_{J}=+1/2\;\rangle$ and $\;|\;↑\;\rangle \equiv |\;L=2,J=5/2,M_{J}=+1/2\;\rangle$ (*43*), with resonant frequency of $\omega_{s}$. The coherent manipulation between the quadrupole transition ∣↓〉 and ∣↑〉 is driven by a narrow-linewidth 729-nm laser, along directions in between the x and y radial axes. This laser is tightly focused for the three- and five-mode implementation, enabling individual addressing for each ion. Figure 1 also illustrates the spectrally resolved motional modes, with frequency $\omega_{i}$ for the *i*th mode. Thus, by simultaneously applying a combination of laser field with frequency components near $\omega_{s}\pm \omega_{i}$ and programmed strengths, the collective spin motion coupling is implemented with Hamiltonians of the blue-sideband (bsb) ${\hat{H}}_{i}^{\text{bsb}}=\frac{\Omega_{i,j}^{\text{bsb}}}{2}{\hat{\sigma }}_{j}^{+}{\hat{a}}_{i}^{†}+h.c$ and red sideband (rsb) ${\hat{H}}_{i}^{\text{rsb}}=\frac{\Omega_{i,j}^{\text{rsb}}}{2}{\hat{\sigma }}_{j}^{+}{\hat{a}}_{i}+h.c.$, for the *j*th spin and *i*th mode. Then, the ${\hat{H}}_{i}^{-}$ in Eq. 3 can be obtained from the desired squeezing parameter r and the number of modes N and implemented by combinations of the required sideband strengths via applying respective laser field frequency components and strengths. Those laser fields are programmed via an acousto-optic modulator, driven by combined radio frequency tones with amplitudes calibrated in separate experiments. The dissipative reset of the spin state to ∣↓〉 is achieved by optical pumping with laser fields of 397, 854, and 866 nm (*43*). In the experimental sequence, each mode is first initialized by Doppler cooling and electromagnetically induced transparency (EIT) cooling (*44*, *45*) to reach a sub-Doppler thermal state, so that the system is in the Lamb-Dicke regime (*46*) and the dynamics of the laser sidebands follow closely the above descriptions. Then, the sequence of dissipative engineering is applied, as shown in Fig. 1B. After cycles of pumping, the system ideally reaches a steady ground state ∣ψ〉 as the global ground state with respect to all transformed annihilation operators ${\hat{K}}_{i}$.

We characterize the motional state by applying a time-varied mapping of the motional information to the spins for state characterization, followed by spin population measurements and fitting of the resulting temporal curve. This mapping is generally achieved by applying an analysis coupling $H_{a}$ jointly to the spins and motion. We perform three types of measurements: (i) We measure populations of the produced state in the original Fock and the Bogoliubov transformed basis (*13*, *27*), denoted as $\left\{\;|\;n_{i}\;\rangle \;\right\}$ and $\{\;|\;n_{i}^{\prime }\;\rangle \equiv \hat{U}|\;n_{i}\;\rangle \;\}$, with ${\hat{H}}_{a}={\hat{H}}_{i}^{\text{bsb}}$ and ${\hat{H}}_{a}={\hat{H}}_{i}^{+}\equiv \frac{\Omega_{i,j}^{-}}{2}{\hat{\sigma }}_{j}^{+}{\hat{K}}_{i}^{†}+h.c.$, respectively, as a generalization of the characterization method for single mode (*27*). We measure the joint populations $|n_{1},n_{2},\ldots \;\rangle$ using additional auxiliary energy levels within the ^40^Ca^+^ ions or additional ions to achieve the independent readout channels for the modes. The latter populations in the transformed basis are denoted as $P\left(n_{i^{\prime }}\right)$. (ii) We estimate the fidelity between the produced state and the desired ∣ψ〉 by $\tilde{F}=\Pi_{i^{\prime }}P\left(0_{i^{\prime }}\right)$ as the multiplication of possibility in the ground state for each transformed basis. Our estimate matches the true fidelity when the produced state is separable in this basis and otherwise gives a lower bound. (iii) We perform correlation measurements between the modes. In particular, we use the van Loock-Furusawa inseparability criteria (*38*–*40*) to verify entanglement. For an N-mode entangled state, it is sufficient to satisfy N−1 inseparability inequalities (ћ=1): $\begin{array}{c} \langle {\left[\Delta \left({\hat{x}}_{1}+{\hat{x}}_{2}+\ldots +{\hat{x}}_{N}\right)\right]}^{2}\rangle +\langle {\left[\Delta \left({\hat{p}}_{m+1}-{\hat{p}}_{m}\right)\right]}^{2}\rangle <2 \end{array}$, where ${\hat{x}}_{j}=\frac{1}{\sqrt{2}}\left({\hat{a}}_{j}+{\hat{a}}_{j}^{†}\right)$, ${\hat{p}}_{j}=\frac{i}{\sqrt{2}}\left({\hat{a}}_{j}^{†}-{\hat{a}}_{j}\right)$, and m=1,2,…,N−1. For the two-mode squeezed state, it is the same as the Einstein-Podolsky-Rosen (EPR) type criteria $\Delta_{\text{EPR}}=\langle {\left[\Delta \left({\hat{x}}_{1}+{\hat{x}}_{2}\right)\right]}^{2}\rangle +\langle {\left[\Delta \left({\hat{p}}_{2}-{\hat{p}}_{1}\right)\right]}^{2}\rangle <2$ (*47*). For the unbiased squeezed state, it is sufficient to only measure the second-order variance $\langle {\left({\hat{x}}_{1}+{\hat{x}}_{2}+\ldots +{\hat{x}}_{N}\right)}^{2}\rangle$ and $\langle {\left({\hat{p}}_{m+1}-{\hat{p}}_{m}\right)}^{2}\rangle$; thus, the inseparability inequalities becomes

$$\Delta_{N,m}=\langle {\left({\hat{x}}_{1}+{\hat{x}}_{2}+\ldots +{\hat{x}}_{N}\right)}^{2}\rangle +\langle {\left({\hat{p}}_{m+1}-{\hat{p}}_{m}\right)}^{2}\rangle <2$$ (4)

which is a stronger bound since in general $\langle {\hat{A}}^{2}\rangle \geq \langle \Delta \hat{A}\rangle$. This variance measurement is implemented by coupling the motional state to a spin via time-varying spin-dependent displacements ${\hat{U}}_{p}=\text{exp}\left(-\mathrm{ik}\hat{A}{\hat{\sigma }}_{x}\right)$, where $k=\Omega_{p}t/2$, $\hat{A}=\left\{({\hat{x}}_{1}+{\hat{x}}_{2}+\ldots +{\hat{x}}_{N}),({\hat{p}}_{m+1}-{\hat{p}}_{m})\right\}$, and $\Omega_{p}$ is the Rabi frequency. For initial spin state ∣↓〉 after applying the time-varying spin-dependent displacement, subsequent spin measurement gives $\begin{array}{c} \langle \hat{O}\left(k\right)\rangle =\langle {\hat{U}}_{p}^{†}{\hat{\sigma }}_{z}{\hat{U}}_{p}\rangle =\langle \text{sin}\left(k\hat{A}\right){\hat{\sigma }}_{y}\rangle +\langle \;\text{cos}\left(k\hat{A}\right){\hat{\sigma }}_{z}\;\rangle \end{array}$. Then, we perform a detection of the spin along ${\hat{\sigma }}_{z}$, and a subsequent quadratic polynomial fit to the spin population curve gives the desired components for the variance ${\frac{d^{2}}{d^{2}k}\left.\langle \hat{O}\left(k\right)\rangle \right|}_{t=0}\propto \langle {\hat{A}}^{2}{\hat{\sigma }}_{z}\rangle$ (*48*, *49*). More details are provided in Materials and Methods.

**Fig. 2.**
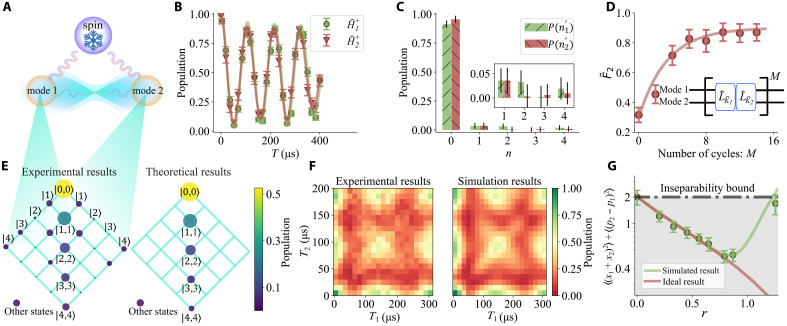
Experimental results of the dissipative preparation of two-mode squeezed state with squeezed parameter r=0.79. (**A**) Two modes coupled with a single dissipative spin. (**B**) Measurements of the steady state of the dissipative process for M=10 dissipative cycles by applying the transformed blue sideband ${\hat{H}}_{i}^{+}$ for a varied duration *T*. (**C**) Derived mode populations $n_{\left\{i=1,2\right\}}$ in the transformed basis from (**D**) via curve fitting, which indicates strong collective phase coherence between the modes. We show the same results for n>0 in the inset for better visibility. (**D**) State fidelity estimate ${\tilde{F}}_{2}$ varied with the number of cycles M. Starting from the thermal state with ${\bar{n}}_{1,2}<0.2$ for both modes, the system reaches the steady state after 10 cycles. (**E**) Measured mode populations in the Fock state basis. The population is determined by model fitting of the data in (**F**). The total population of the unmeasured states is estimated, depicted on the side of the grids labeled as “other states.” The area and color of the circles represent the fitted population on each basis. The population is mainly diagonal, indicating a strong two-body correlation of the modes. (F) Experimental results of the spin population evolution under the blue sideband ${\hat{H}}_{i}^{\text{bsb}}$ in the Fock basis compared with simulation results. (**G**) EPR-type variance $\langle {\left({\hat{x}}_{1}+{\hat{x}}_{2}\right)}^{2}\rangle +\langle {\left({\hat{p}}_{2}-{\hat{p}}_{1}\right)}^{2}\rangle$ varied with the squeezed parameter r, as a witness of the entanglement bound depicted with the black dashed line shows. Entanglement can be identified below such a bound with $\langle {\left({\hat{x}}_{1}+{\hat{x}}_{2}\right)}^{2}\rangle +\langle {\left({\hat{p}}_{2}-{\hat{p}}_{1}\right)}^{2}\rangle <2$. The green dots are experimental data, and the error bars are derived from the fitting uncertainties. The red line shows $\langle {\left({\hat{x}}_{1}+{\hat{x}}_{2}\right)}^{2}\rangle +\langle {\left({\hat{p}}_{2}-{\hat{p}}_{1}\right)}^{2}\rangle$ of the ideal two-mode squeezed state. The green line shows the numerical simulation results of M=10 dissipation cycles.

We first demonstrate the generation of a two-mode squeezed state, as shown in Fig. 2A. We trap a single ^40^Ca^+^ ion and operate on the two radial modes with frequencies $\left\{\;\omega_{x},\omega_{y}\;\right\}=2\pi \times \left\{\;1.12,0.90\;\right\}$ MHz. We apply the transformed spin-motion couplings ${\hat{H}}_{i}^{-}$ using the combination of blue sideband and red sideband for r=0.79 corresponding to Eqs. 2 and 3. The resulting spin motion coupling strengths $\Omega^{-}$ are approximately 2π×6.8 kHz, and each coupling is applied for 55 μs. We apply the sequences for M variable repetitions followed by a measurement. For M=10 as an example, Figure 2B shows temporal spin oscillation due to the application of the engineered blue sideband ${\hat{H}}_{i}^{+}$ for varied durations. Such curve contains an excitation-dependent oscillation rate, thus we can extract the excitation populations $P(n_{i}^{\prime })$ by a curve fitting to a model, as shown in Fig. 2C, showing the majority of the populations are in the ground states of ${\hat{K}}_{i}$. The estimated fidelity ${\tilde{F}}_{2}=P(0_{1}^{\prime })P(0_{2}^{\prime })$ is then obtained, as shown in Fig. 2D for varied M. As the number of cycles increases, the system reaches a steady state after approximately M=10 pumping cycles, and we obtain a fidelity estimated to be 86(6)% from this state. We also use three internal states (∣↓〉, ∣↑〉, and $\begin{array}{c} |\;\text{AUX}\;\rangle \equiv |\;L=2,J=5/2,M_{J}=+\;5/2\;\rangle \end{array}$) to measure the joint populations in the basis of $|\;n_{1},n_{2}\;\rangle$. We perform blue sideband transition $|\;↓,n_{1}\;\rangle ↔|\;↑,n_{1}+1\;\rangle$ and $|\;↓,n_{2}\;\rangle ↔|\;\text{AUX},n_{2}+1\;\rangle$ in sequence with variational scanning time $T_{1}$ and $T_{2}$. The two-dimensional (2D) time evolution is shown in Fig. 2F, matching with numerical simulations. The population is obtained by curve fitting of Fig. 2E to a multimode model. We can observe that the majority of populations are in the correlated pairs such as ∣0,0〉, ∣1,1〉, ∣2,2〉_,_ and ∣3,3〉, which agrees well with theoretical predictions of a two-mode squeezed state given r. Full fitting methods are shown in Materials and Methods. From Fig. 2 (C and E), we can imply that the produced state closely matches with the desired one in terms of the mode populations in the original and transformed bases. Regarding the entanglement feature of the produced state, we give a quantitative analysis in the following.

The genuine multipartite entanglement of the two-mode squeezed state is analyzed by measuring $\Delta_{N,m}$, described above and in the Supplementary Materials, depicted in Fig. 2G with varied squeezing parameters r for M=10 dissipation cycles under the same Rabi frequency in the transformed basis. We observe $\Delta_{2,1}$ saturates near r=0.8, which matches our model considering experimental imperfections (see Supplementary Materials). In particular, with r=0.79, we obtain $\Delta_{2,1}=0.43\pm 0.06$, which is close to the ideal state value of $\Delta_{2,1}=0.41$, satisfying entanglement criteria $\Delta_{2,1}<2$. Deviations from the ideal cases with larger squeezing values (r>1.0) are due to the technical limitations in our apparatus with a maximal amount of pulses, leading to a state that has not yet reached the steady state. In the absence of unanticipated additional noise sources, these deviations could be alleviated by adding more dissipation cycles, as predicted in the theoretical analysis (see the Supplementary Materials for details).

**Fig. 3.**
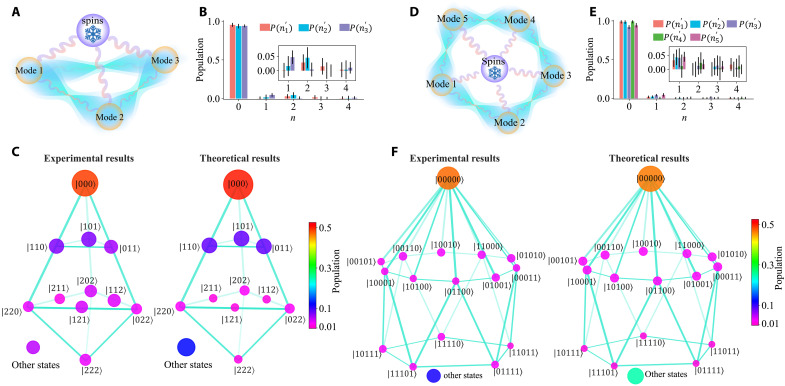
Experimental results of the stabilized multimode squeezed state. (**A** to **C**) Three modes coupled with spins and (**D** to **F**) five modes coupled with spins. [(A) and (D)] Connections of spins with the modes. [(B) and (E)] Derived mode populations in the transformed basis from fitting the blue sideband ${\hat{H}}_{i}^{+}$. The resulting fidelities are 84(4)% and 84(6)%, respectively. We show the same results for n>0 in the inset for better visibility. [(C) and (F)] Mode population in the Fock state basis. The grid describes the Hilbert space of the system that consists of all the vibrational modes. The labeled nodes are the states with the population larger than 1%. Other states are the total population that are not included in the grid. The circles’ area and color represent the fitted population in each basis, showing two-body correlations. We observe superpositions of pairwise excitation, as expected for multimode squeezed state $\text{exp}\left[\frac{r}{2}\sum_{i,j}G_{i,j}\left({\hat{a}}_{i}{\hat{a}}_{j}-{\hat{a}}_{i}^{†}{\hat{a}}_{j}^{†}\right)\right]|\;0^{⊗N}\;\rangle$ in the text.

We further trap two ions to demonstrate the three-mode squeezed state with r=0.5 using modes with frequencies $\left\{\omega_{x1},\omega_{x2},\omega_{y1}\right\}=2\pi \times \left\{1.12,0.98,0.90\right\}$ MHz. Similar to the two-mode case, we use the spin of the first ion coupled with multiple modes to create entanglement using a tightly focused 729-nm laser, as illustrated in Fig. 3A. We apply 100-μs spin-motion coupling, with strengths $\Omega^{-}$ of 2π×4.4 kHz. After applying the dissipation sequence described above for 10 cycles, we measure the populations $P(n_{i}^{\prime })$ using the addressed transformed blue sideband and obtain an estimation of fidelity ${\tilde{F}}_{3}=84\left(4\right)\%$, derived from the multiplications of measured populations in the transformed basis as shown in Fig. 3B. We also analyze the state in the Fock state basis by performing addressed blue sideband fitting using four internal states, as shown in Fig. 3C, similar to the two-mode implementation described above. We observe that most of the populations in the Fock state basis are in a two-body correlated pattern, and the population results agree well with our theoretical expectations. We further measure the correlation variances to check the entanglement, summarized in Table 1. $\begin{array}{c} \left\{\;\Delta_{3,1},\Delta_{3,2}\;\right\}=\left\{\;0.60\pm 0.21,0.64\pm 0.24\;\right\} \end{array}$ are all well below the criteria, which indicates the genuineness of the multipartite entanglement.

**Table 1.** Inseparability of entangled modes. Measured Loock-Furusawa–type inseparability parameters (as in Eq. 4) for various squeezed states. All measured values are below 2 (ℏ=1), indicating entanglement.

State	Inseparable parameters	r	Measured value
Two mode	$\langle {\left({\hat{x}}_{1}+{\hat{x}}_{2}\right)}^{2}\rangle +\langle {\left({\hat{p}}_{2}-{\hat{p}}_{1}\right)}^{2}\rangle$	0.79	0.42±0.06
Three mode	$\langle {\left({\hat{x}}_{1}+{\hat{x}}_{2}+{\hat{x}}_{3}\right)}^{2}\rangle +\langle {\left({\hat{p}}_{2}-{\hat{p}}_{1}\right)}^{2}\rangle$	0.5	0.60±0.21
	$\langle {\left({\hat{x}}_{1}+{\hat{x}}_{2}+{\hat{x}}_{3}\right)}^{2}\rangle +\langle {\left({\hat{p}}_{3}-{\hat{p}}_{2}\right)}^{2}\rangle$		0.64±0.24
Five mode	$\langle {\left({\hat{x}}_{1}+\cdots +{\hat{x}}_{5}\right)}^{2}\rangle +\langle {\left({\hat{p}}_{2}-{\hat{p}}_{1}\right)}^{2}\rangle$	0.3	1.07±0.22
	$\langle {\left({\hat{x}}_{1}+\cdots +{\hat{x}}_{5}\right)}^{2}\rangle +\langle {\left({\hat{p}}_{3}-{\hat{p}}_{2}\right)}^{2}\rangle$		1.03±0.21
	$\langle {\left({\hat{x}}_{1}+\cdots +{\hat{x}}_{5}\right)}^{2}\rangle +\langle {\left({\hat{p}}_{4}-{\hat{p}}_{3}\right)}^{2}\rangle$		1.17±0.29
	$\langle {\left({\hat{x}}_{1}+\cdots +{\hat{x}}_{5}\right)}^{2}\rangle +\langle {\left({\hat{p}}_{5}-{\hat{p}}_{4}\right)}^{2}\rangle$		1.25±0.19

To demonstrate scalability, we trap five ions in a chain and prepare the five-mode squeezed state with r=0.3, as illustrated in Fig. 3D. We choose five frequency-resolved motional modes in the radial direction with frequencies $\begin{array}{c} \left\{\;\omega_{x1},\omega_{x2},\omega_{x3},\omega_{x4},\omega_{y1}\;\right\}=2\pi \times \left\{\;1.65,1.61,1.54,1.46,1.31\;\right\} \end{array}$ MHz, as shown in Fig. 1D. The second ion has a relatively large coupling strength with all these modes, so we apply the spin-motion coupling with this ion using the addressed 729-nm laser. We apply 170-μs transformed spin motion coupling, with Rabi strengths $\Omega_{2}^{-}$ of 2π×3.0 kHz. After applying the dissipation sequence described above for 10 cycles, we measure the populations $P(n_{i}^{\prime })$ using the addressed transformed blue sideband of the second ion. We then obtain an estimation of fidelity ${\tilde{F}}_{5}=84\left(6\right)\%$, derived from the multiplications of measured populations in the transformed basis as shown in Fig. 3E. We also analyze the state in the Fock state basis using each ion as an independent readout channel. To achieve this, we apply an addressed red sideband transition on each ion, coupling it to a specific motional mode. This process maps the motional state onto the internal state of the ion, enabling site-resolved detection via electron-multiplying charge-coupled device (EMCCD) (see Materials and Methods). The population in the Fock basis agrees well with the simulation results, as shown in Fig. 3F. We further evaluate the correlation variance, as presented in Table 1. $\left\{\Delta_{5,1},\Delta_{5,2},\;\Delta_{5,3},\;\Delta_{5,4}\right\}=\left\{1.07\pm 0.22,1.03\pm 0.21,1.17\pm 0.29,1.25\pm 0.19\right\}$, which are below the criteria, suggesting the genuineness of the multipartite entanglement.

## DISCUSSION

In summary, we demonstrate programmable dissipative engineering for entangled state production with two, three, and five bosonic modes in vibrating trapped ions. Taking advantage of precise control over the coupling between multiple motional modes and the internal state of ions, we can scale our system to accommodate a larger number of ions and motional modes. Based on our demonstration, using focused lasers to address individual ions simultaneously would enable parallel pumping for each collective mode with more versatile control. Our scheme can also be generalized to achieve multimode Gaussian states in other experimental platforms, for example, superconducting cavities, atomic ensembles, and nanomechanics, depending on the system’s connectivity. Because the multimode squeezed states are widely used as key resources for measurement-based quantum computation (*50*, *51*) and multiparameter estimation (*52*, *53*), stable generation of these states would bring in unique properties such as event-ready entangled source and insensitivity to drive strength fluctuations. Our work currently relies on driving sideband in the linear regime; however, recent demonstrations of effective high-order operators compiled from low-order drivings (*54*) would enable dissipative generation of motional modes with nonlinear couplings. This application may also help generate non-Gaussian states for applications such as cat codes (*55*).

## MATERIALS AND METHODS

### Experimental details

In this work, we use trapped ^40^Ca^+^ ions in a linear Paul trap (*56*) as shown in fig. S2. For the two-mode squeezed state, we use the platform with only global manipulation and detection. The trap frequencies of the center of mass (COM) mode are the same in these two experiments, with $\omega_{x}=2\pi \times 1.12$ MHz, $\omega_{y}=2\pi \times 0.90$ MHz, $\omega_{z}=2\pi \times 0.54$ MHz in the three directions. The COM mode frequencies are measured by applying electric fields with varied frequencies to find the resonance that can efficiently excite the motional modes, known as “tickle” spectroscopy, with uncertainty less than 50 Hz. The other mode frequencies are measured by Ramsey-like experiment with displacement operation as shown in (*57*). Before each experiment, these modes are initialized by Doppler cooling and EIT cooling, as shown in fig. S2. Doppler cooling is performed via an 866-nm laser resonant with the dipole transitions between $|D_{3/2}\rangle$ and $|P_{1/2}\rangle$, and a 397-nm laser near detuned to the dipole transitions between $|S_{1/2}\rangle$ and $|P_{1/2}\rangle$. A pair of far blue detuned 397-nm lasers are used for EIT cooling in the radial direction. After EIT cooling, the mean phonon number of these modes can be cooled to less than 0.2. The quadrupole transition between $|S_{1/2}\rangle$ and $|D_{5/2}\rangle$ is used for coherent manipulations driven by a narrow linewidth of 729-nm laser. We use the global 729-nm lasers along the radial direction, and the Lamb-Dicke factors of one ion’s radial modes are $\eta_{x}=0.06$ and $\eta_{y}=0.07$, respectively, which means that the experiments are well described by the Lamb-Dicke approximation with η≪1 (*56*). We also measure the heating rate of the two radial COM modes with one ion as shown in fig. S3. The heating rates of a single ion for modes 1 and 2 are 8(3) and 25(3) phonon/s, respectively, which is low enough for our motional state control. The heating rate of the stretch mode for two ions is much lower, which can be neglected during the experiment. The coherence time of these modes is about 8 ms. The internal electronic state of the ion is initialized to ∣↓〉 by a combination of $\sigma^{+}$ polarized 397-nm laser and linearly polarized 866-nm and 854-nm lasers. We can discriminate internal state using fluorescence detection with the 397-nm cycling transition between $|S_{1/2}\rangle$ and $|P_{1/2}\rangle$ and an auxiliary 866-nm optical pumping transition (*58*).

For the three-mode and five-mode squeezed state, we use the upgraded platform with the ability of single-qubit addressing and site-resolved detection. The trapping frequencies of the COM mode with two ions for three-mode squeezed state in the three directions are $\omega_{x}=2\pi \times 1.12$ MHz, $\omega_{y}=2\pi \times 0.90$ MHz, and $\omega_{z}=2\pi \times 0.54$ MHz. We choose three radial mode with frequencies $\{\omega_{1},\omega_{2},\omega_{3}=2\pi \times \left\{\;1.12,0.98,0.90\right\}$ MHz. The trapping frequencies of the COM mode with five ions in the three directions are $\omega_{x}=2\pi \times 1.65$ MHz, $\omega_{y}=2\pi \times 1.31$ MHz, and $\omega_{z}=2\pi \times 0.37$ MHz. We choose the modes in radial direction with frequencies $\left\{\omega_{x1},\omega_{x2},\omega_{x3},\omega_{x4},\omega_{y1}\right\}=2\pi \times \left\{1.65,1.61,1.54,1.46,1.31\;\right\}$ MHz. These five modes are chosen because of their frequency stability (peak-to-peak value of half-hour long drift less than 100 Hz) and are discrete in the frequency domain. The heating rate for COM mode $\omega_{x1}$ is 3(1) phonon/s and $\omega_{y1}$ is 5(3)phonon/s, respectively, and the heating rates of other modes are much lower than COM mode. The coherence time of these modes is about 4 ms. The cooling is the same as before, and all the modes are cooled to about 0.1 mean phonon number after the EIT cooling. We use the addressed 729-nm laser along the radial direction to individually control the spins. The laser’s direction is controlled by the two-dimensional acousto-optical deflectors and is focused to 2.5 μm waist so that the cross-talk in terms of Rabi frequency of the nearby spins is less than 3%. The modes we used have relatively large Lamb-Dicke parameters with the second ion, so we choose this ion as the reservoir to perform the dissipative preparation. The detection laser of the spins is the same as two- and three-mode cases, and we use EMCCD instead of photomultiplier tube for site-resolved detection. The detection time is about 3.5 ms, so the state preparation and measurement (SPAM) error for each ion is about 0.3%.

### Sufficient condition for genuine N-partite entanglement

Here we use the van Loock-Furusawa inseparability criteria (*38*) to verify entanglement. This includes the measurement of mode correlation variances, which is done by the spin-dependent displacement. The typical spin evolution for the displacement along $\hat{A}=\left\{\;({\hat{x}}_{1}+{\hat{x}}_{2}+\ldots {\hat{x}}_{N}),({\hat{p}}_{m+1}-{\hat{p}}_{m})\;\right\}$ is shown in fig. S4. We use the quadratic polynomial fit $\langle {\hat{\sigma }}_{z}\rangle ={\mathrm{ak}}^{2}+\mathrm{bk}+c$ to the spin population curve to give the desired components for the variance $\langle {\hat{A}}^{2}\rangle =2a$. The corresponding inseparability parameter is detailed in Table 1.

### Two-mode squeezed state

Here, we show the original fitting results of the two-mode squeezed state population in the Fock basis and the engineered basis, which is shown in Fig. 2.

We characterize the two-mode squeezed state with two different methods. First, we can obtain the fidelity after the dissipative process by the fitting results of blue sideband Rabi flopping of Bogoliubov operators, respectively, with the Hamiltonian written as ${\hat{H}}_{1,2}^{+}=\Omega^{+}/2\left({\hat{K}}_{1,2}{\hat{\sigma }}^{-}+{\hat{K}}_{1,2}^{†}{\hat{\sigma }}^{+}\right)$. The population of the state ∣↓〉 can be written as a function of the blue sideband pulse duration t

$$\begin{array}{c} P_{1,2}\left(\;|\;↓\;\rangle \right)=\frac{1}{2}\sum_{n}p_{1,2}\left(n\right)\left[1+e^{-\gamma_{n}t}\text{cos}\left(\Omega_{n}t\right)\right] \end{array}$$ (5)

where $p_{K}\left(n\right)$ is the population of the nth energy eigenstate, $\Omega_{n}$ is the Rabi frequency for the transtion between $|\;↓\;\rangle ,\hat{U}\;|\;n\;\rangle$ and $|\;↑\;\rangle ,\hat{U}\;|\;n+1\;\rangle$ and $\gamma_{n}$ describe the decoherence of ions and laser. We can obtain the population $p_{1,2}\left(n\right)$ by fitting the formula Eq. 5, as shown in Fig. 2 (B and C).

We also use another method to verify these states independently. For the two-mode squeezed state, we use three internal states (∣↓〉, ∣↑〉, and $\begin{array}{c} |\;\text{AUX}\;\rangle \equiv |\;L=2,J=5/2,M_{J}=+\;5/2\rangle \end{array}$) to measure the populations in the Fock basis. We perform the sequential blue sideband Hamiltonian written as ${\hat{H}}_{1,2}^{+}=\Omega /2\left({\hat{a}}_{1,2}{\hat{\sigma }}^{-}+{\hat{a}}_{1,2}^{†}{\hat{\sigma }}^{+}\right)$. The population of the state ∣↓〉 can be written as a function of duration $t_{1}$ and $t_{2}$ of the two blue sideband pulse, respectively

$$\begin{array}{c} \begin{array}{c} P\left(|\;↓\;\rangle \right)=\frac{1}{4}\sum_{n,m}p\left(n,m\right)\left\{\;\left[1+e^{-\gamma_{n}t_{1}}\text{cos}(\left.\Omega_{n}t_{1})\right]\;\right.\right\}\\ \left[1+e^{-\gamma_{m}t_{2}}\text{cos}\left(\Omega_{m}t_{2}\right)\right] \end{array} \end{array}$$ (6)

where p(n,m) is the population of the nth energy eigenstate of mode 1 and mth energy eigenstate of mode 2. We can obtain the population p(n,m) by fitting each set of data by the Eq. 6.

### Three-mode squeezed state

For the three-mode squeezed states case, we use three collective vibrational modes in radial directions of a two-ion chain and the internal state of the first ion as the reservoir. Same as before, the three-mode squeezed state is the joint ground state of the three Bogoliubov operators. We can use the engineered blue sideband to extract the fidelity of the process, with the spin motion coupling Hamiltonians written as

$${\hat{H}}_{1,2,3}^{+}=\frac{\Omega_{1}^{+}}{2}\left({\hat{K}}_{1,2,3}{\hat{\sigma }}_{1}^{-}+{\hat{K}}_{1,2,3}^{†}{\hat{\sigma }}_{1}^{+}\right)$$ (7)

Then, the population of the first spin $|\;↓\;\rangle_{1}$ can be written as a function of the blue sideband pulse duration t

$$\begin{array}{c} P_{1,2,3}\left(|\;↓\;\rangle_{1}\right)=\frac{1}{2}\sum_{n}p_{1,2,3}\left(n\right)\left[1+e^{-\gamma_{n}t}\text{cos}\left(\Omega_{n}t\right)\right] \end{array}$$ (8)

We can obtain the population $p_{i}\left(n\right)$ with i=1,2,3 by fitting each set of data by the Eq. 8. The Rabi flopping and the fitting results are shown in fig. S6, with the lower bound of fidelity of the state being 84(4)%.

We also verify the population in the Fock state basis. For the three-mode squeezed states, we need three transitions to obtain all the population information. Specifically, ∣↓〉, ∣↑〉, $|{\text{AUX}}_{1}\rangle$, and $\begin{array}{c} |\;{\text{AUX}}_{2}\;\rangle \equiv |\;L=2,J=5/2,M_{J}=-3/2\rangle \end{array}$ are used. We perform three blue sideband transtions ∣↓,n〉↔∣↑,n+1〉, $|\;↓,m\;\rangle ↔|\;{\text{AUX}}_{1},m+1\;\rangle$, and $|\;↓,l\;\rangle ↔|\;{\text{AUX}}_{2},l+1\;\rangle$ in sequence. The population of the first spin $|\;↓\;\rangle_{1}$ can be written as a function of duration $t_{1}$, $t_{2}$, and $t_{3}$ of the three blue sideband pulse, respectively

$$\begin{array}{c} \begin{array}{cc} P\left(|\;↓\;\rangle_{1}\right)=\frac{1}{8}\sum_{n,m,l}p\left(n,m,l\right) & \left[1+e^{-\gamma_{n}t_{1}}\text{cos}\left(\Omega_{n}t_{1}\right)\right]\left[1+e^{-\gamma_{m}t_{2}}\text{cos}\left(\Omega_{m}t_{2}\right)\right]\left[1+e^{-\gamma_{l}t_{3}}\text{cos}\left(\Omega_{l}t_{3}\right)\right] \end{array} \end{array}$$ (9)

where p(n,m,l) is the population of the nth energy eigenstate of mode 1, mth energy eigenstate of mode 2, and lth energy eigenstate of mode 3. We can obtain the population p(n,m,l) by fitting each dataset by Eq. 9. The population fitted in the Fock state basis and Bogoliubov basis is all shown in fig. S6.

### Five-mode squeezed state

For the five-mode squeezed states case, we use five collective vibrational modes in radial directions of a five-ion chain and use the internal state of the second ion as the reservoir. Same as before, the five-mode squeezed state is the joint ground state of the five Bogoliubov operators. The blue sideband of Bogoliubov operators of the second ion is

$${\hat{H}}_{1,2,3,4,5}^{+}=\frac{\Omega_{2}^{+}}{2}\left({\hat{K}}_{1,2,3,4,5}{\hat{\sigma }}_{2}^{-}+{\hat{K}}_{1,2,3,4,5}^{†}{\hat{\sigma }}_{2}^{+}\right)$$ (10)

The Rabi flopping and the fitting results are shown in fig. S7. We can obtain the population $p_{i}\left(n\right)$ with i=1∼5 by fitting each set of data using the equation

$$\begin{array}{c} P_{1∼5}\left(|\;↓\;\rangle_{2}\right)=\frac{1}{2}\sum_{n}p_{1∼5}\left(n\right)\left[1+e^{-\gamma_{n}t}\text{cos}\left(\Omega_{n}t\right)\right] \end{array}$$ (11)

The state fidelity is estimated as 84(6)%.

We also verify the population in the Fock state basis. The blue sideband fitting method is too time-consuming for the five-mode cases as we need to scan 5^20^ points with similar accuracy as the three-mode case, which is more than 2000 times. To characterize the mode population with time efficiency, we use each ion as an independent readout channel to map each mode onto each spin and read them simultaneously using EMCCD, which enables phonon correlation detection. Suppose that the final phonon state’s population is within (0,1) subspace, and we want to determine all these populations, as the simulation indicates that the other states’ maximum occupations are about 0.1%, smaller than our SPAM error (~0.3%). We apply the red sideband of each ion with each mode one by one in sequence with the coupling time equal to the π time of ∣↓,1〉→∣↑,0〉 and readout the spins’ population. When one spin is in the bright state ∣↓〉, indicating the corresponding phonon is in the ground state ∣0〉, while the phonon state is ∣1〉 if the related spin is in the dark state ∣↑〉. For instance, if the phonon state is ∣00011〉, then it will evolve to ∣↓↓↓↑↑〉. Thus, the site-resolved detection leads to the single-shot readout of all the modes in the Fock state basis, as shown in fig. S7B.

## Supplementary Materials

### This PDF file includes:

Supplementary Text Figs. S1 to S7 References
