# Supplementary material for: Programmable multi-mode entanglement via dissipative engineering in vibrating trapped ions
Source: Sci Adv. 2025 Jul 2;11(27):eadv7838. doi: 10.1126/sciadv.adv7838 (PMC13155508; doi:10.1126/sciadv.adv7838)
Supplement: Supplementary file 1 — Supplementary Text Figs. S1 to S7 References [file sciadv.adv7838_sm.pdf]

Science Advances
AAAS

Supplementary Materials for

Programmable multi-mode entanglement via dissipative engineering in vibrating trapped ions

Yue Li *et al.*

Corresponding author: Yiheng Lin, yiheng@ustc.edu.cn

Sci. Adv. **11**, eadv7838 (2025)
DOI: 10.1126/sciadv.adv7838

This PDF file includes:

Supplementary Text
Figs. S1 to S7
References

Supplementary Text

Theoretical derivation of the dissipative preparation

We first give a brief introduction to Gaussian operations and Gaussian states (*59*). Considering N bosonic modes with the annihilation and creation operators $\{\hat{a}_i, \hat{a}_i^\dagger\}_{i=1}^N$. These operators can be arranged into a vectorial operator $\hat{\mathbf{a}} = (\hat{a}_1, \hat{a}_2, ..., \hat{a}_N)$ and $\hat{\mathbf{a}}^\dagger = (\hat{a}_1^\dagger, \hat{a}_2^\dagger, ..., \hat{a}_N^\dagger)$. Gaussian states are generated by applying the Gaussian unitaries to the thermal state. A generic unitaries, $\hat{U} = \exp(-i\hat{H}/2)$, are derived from a quadratic Hamiltonian $\hat{H} = i(\hat{\mathbf{a}}^\dagger\boldsymbol{\alpha} + \hat{\mathbf{a}}^\dagger\mathbf{F}\hat{\mathbf{a}}^T + \hat{\mathbf{a}}^\dagger\mathbf{G}\hat{\mathbf{a}}^{\dagger T}) + h.c.$, where $\boldsymbol{\alpha} \in \mathbb{C}^N$, $\mathbf{F}$ and $\mathbf{G}$ are $N \times N$ complex matrices correspond to the correlation between the modes. In the Heisenberg picture, this kind of unitary corresponds to a linear unitary Bogoliubov transformation, resulting in transformed annihilation operators $\hat{\mathbf{K}} = (\hat{K}_1, \hat{K}_2, ..., \hat{K}_N)$

$$\hat{\mathbf{a}} \to \hat{\mathbf{K}} \equiv \hat{U}\hat{\mathbf{a}}\hat{U}^\dagger = \hat{\mathbf{a}}\mathbf{A} + \hat{\mathbf{a}}^\dagger\mathbf{B} + \boldsymbol{\alpha}, \tag{S1}$$

where $\mathbf{A}$ and $\mathbf{B}$ are $N \times N$ complex matrices satisfying $\mathbf{A}\mathbf{B}^T = \mathbf{B}\mathbf{A}^T$ and $\mathbf{A}\mathbf{A}^\dagger = \mathbf{B}\mathbf{B}^\dagger + \mathbf{I}$ with $\mathbf{I}$ is the identity matrix, while $\hat{U}$ is a Gaussian unitary. Under these transformed bases, the ground state is simply the pure state corresponding to acting the Gaussian unitaries onto the vacuum state. Thus, by engineering the environment to change the correlation between the motional modes, we can cool the system to the desired ground state. The transformed spin-motion coupling can be implemented with Hamiltonians of the *blue-sideband* $\hat{H}_i^{bsb} = \frac{\Omega_{i,j}^{bsb}}{2}\hat{\sigma}_j^+\hat{a}_i^\dagger + h.c.$, *carrier* $\hat{H}_i^{carrier} = \frac{\Omega_{i,j}^{carrier}}{2}\hat{\sigma}_j^+ + h.c.$, *red-sideband* $\hat{H}_i^{rsb} = \frac{\Omega_{i,j}^{rsb}}{2}\hat{\sigma}_j^+\hat{a}_i + h.c.$, for the jth spin and i^{th} mode.

Here we consider the multi-mode squeezed state with the unitary operator written as

$$\hat{U} = \exp\left[\frac{\xi}{2}\sum_{i,j} G_{i,j}(\hat{a}_i\hat{a}_j - \hat{a}_i^\dagger\hat{a}_j^\dagger)\right], \tag{S2}$$

where $\xi = re^{i\phi}$ is the squeezing parameter and $\mathbf{G}$ is the adjacency matrix that encodes the connectivity of the modes. Without loss of generality, we set the phase factor $\phi = 0$. For the all-to-all connected N-mode squeezed state, the adjacency matrix becomes $\mathbf{G} = \mathbb{I} - I$, where $\mathbb{I}$ is the matrix with all elements equal to 1 and I is the identity matrix. Applying the Bogoliubov

transformation, the transformed annihilation operator is written as

$$\begin{aligned}
\hat{K}_i &= \sum_k (\mathbf{G}^0)_{i,k}\hat{a}_k - r\sum_k (\mathbf{G}^1)_{i,k}\hat{a}_k^\dagger \\
&+\frac{1}{2!}r^2\sum_k \left(\mathbf{G}^2\right)_{i,k}\hat{a}_k - \frac{1}{3!}r^3\sum_k \left(\mathbf{G}^3\right)_{i,k}\hat{a}_k^\dagger + \ldots \\
&= \left\{\frac{1}{N}\cosh\left[(N-1)r\right] + \frac{N-1}{N}\cosh(r)\right\}\hat{a}_i \\
&+ \sum_{k\neq i}\left\{\frac{1}{N}\cosh\left[(N-1)r\right] - \frac{1}{N}\cosh(r)\right\}\hat{a}_k \\
&+ \left\{\frac{1}{N}\sinh\left[(N-1)r\right] - \frac{N-1}{N}\sinh(r)\right\}\hat{a}_i^\dagger \\
&+ \sum_{k\neq i}\left\{\frac{1}{N}\sinh\left[(N-1)r\right] + \frac{1}{N}\sinh(r)\right\}\hat{a}_k^\dagger \\
&\equiv \sum_k A_{i,k}\hat{a}_k + B_{i,k}\hat{a}_k^\dagger
\end{aligned} \tag{S3}$$

Here we use the equation $\mathbf{G}^n = \frac{(N-1)^n-(-1)^n}{N}\mathbb{I} + (-1)^n I$. Then the Rabi frequencies of the sideband can be derived from the **A** and **B** matrix.

Scaling to larger number of modes

In this section, we consider how our approach could be applied to systems with a larger mode number N. In the following, we discuss potential challenges in terms of long required pulse durations and motional mode frequency crowding for large N. We also give possible solutions with respect to each of these issues.

We first discuss the scaling requirements for pulse duration. Equation 4 of the main text shows the amplitudes of the sideband drives after Bogoliubov transformation. Thus, the total effective intensity for one engineered sideband scales as $\sum_k A_{i,k}^2 + B_{i,k}^2 \leq \left(\sum_k A_{i,k} + B_{i,k}\right)^2 \sim e^{(N-1)r}$. For a limited laser intensity, it would in turn take exponentially long duration scaling as both N. Within this limitation, we are able to generate up to five-mode squeezed states. To scale beyond this, one practical strategy is to independently prepare several five-mode squeezed states and then apply beam splitter (BS) operations between them (*60*), inspired by techniques developed in photonic experiments (*50, 51*). This approach enables the construction of larger multimode entangled states from smaller building blocks with our dissipative method.

The issue of mode crowding is also an important challenge for phonon-based quantum information processing. However, the idea of our approach could still be applicable to medium sized ion chain. Extending to even larger system sizes may require alternative architectures, such as segmented ion chains. In this approach, the desired states could be generated within individual chains, while larger entangled states could be constructed through precise control of each chain and modular, tunable center-of-mass mode couplings between adjacent chains (*61, 62*).

Experimental imperfection

Here we discuss the sources of error in state preparation infidelity. These can be categorized into motional mode decoherence and spin decoherence.

The dominant source of error in our scheme is the mode frequency drift, which is primarily caused by the instability of the radio-frequency (RF) signal generator and resonator. Since we utilize radial modes to generate entangled states, these electronic instabilities lead to mode frequency fluctuations on the order of $2\pi \times 50$ Hz, which affects the stability of the dissipative process and contributes up to an estimated 15% infidelity in the two-mode squeezed state generation. In the following, we introduce the simulation methods to obtain such a quantity. Using two-mode squeezed state as an example, we use $\hat{U}_{1,2} = \exp(-i\hat{H}^{-}_{1,2} \times \frac{\pi}{2\Omega_{1,2}})$ to describe the coherent part. Under this coherent control, the output state is $\hat{\rho}_{out} = \hat{U}_{1,2}\hat{\rho}_{in}\hat{U}^{\dagger}_{1,2}$. After the coherent part, we calculate the spin partial trace of the output state $\hat{\rho}_{motion} = \mathrm{Tr}_{spin}(\hat{\rho}_{out})$ to simulate the pumping process. Then the input state of the next cycle can be written as $\hat{\rho}_{in} = |{\downarrow\downarrow}\rangle \bigotimes \hat{\rho}_{motion}$. The main error resource is motional frequency fluctuation. This can be modeled as the random drift during the coherent control by $\hat{U}_{1,2} = \exp(-i(\hbar\Delta_1\hat{a}^{\dagger}_1\hat{a}_1 + \hbar\Delta_2\hat{a}^{\dagger}_2\hat{a}_2 + \hat{H}_{1,2}) \times \frac{\pi}{2\Omega^{-}_{1,2}})$. Motional phase noise can significantly affect the final squeezing performance, but its impact can be mitigated through: real-time frequency stabilization of the RF source to reduce mode drift; dynamical decoupling techniques to suppress low-frequency noise components affecting the motional modes; active feedback control on the trap voltages to compensate for slow drifts in mode frequencies.

Mode heating is another potential source of infidelity. However, in our setup, the measured heating rate is relatively low (≤ 25 quanta/s), which is small compared to the dissipative cooling rate and thus does not significantly impact the final state.

Unlike unitary gate-based approaches, our dissipative scheme is intrinsically robust to spin decoherence sources such as laser phase noise and magnetic field fluctuations. This is because the spin degrees of freedom only participate in sideband transitions before being optically pumped, meaning that any spin decoherence primarily affects the pumping rate rather than the steady-state dark mode. Thus, while spin decoherence may influence the speed of the dissipative process, it does not significantly degrade the final state fidelity.

Primary sources of error of the variance

Here, we will discuss the primary sources of error in this variance measurement. This error budget includes statistical errors, state preparation and measurement (SPAM) errors, and systematic errors.

The primary source of error arises from statistical errors, which stem from the inherent binomial distribution of the spin measurement and fitting error. With 1000 repetitions per measurement point, Monte Carlo simulations indicate that the resulting statistical uncertainty in $\langle \hat{A}^2 \rangle$ is around $\langle \hat{A}^2 \rangle \times 35\%$, for evolution times during which $\langle \hat{\sigma}_z \rangle$ evolves from -1 to about -0.8.

Next, for the state preparation and measurement error, it is approximately 0.3% per ion, primarily due to the readout time limitations of the EMCCD. For our experiments, this results in less than $\langle \hat{A}^2 \rangle \times 1\%$ error in the determination of $\langle \hat{A}^2 \rangle$.

Finally, we discuss the systematic errors, including Rabi frequency drift and detuning errors. A drift of about 1% in the Rabi frequency contributes an estimated error $\langle \hat{A}^2 \rangle \times 2\%$. The detuning errors are controlled to within 50 Hz, contributing negligibly to the overall error budget $\langle \hat{A}^2 \rangle \times 0.5\%$.

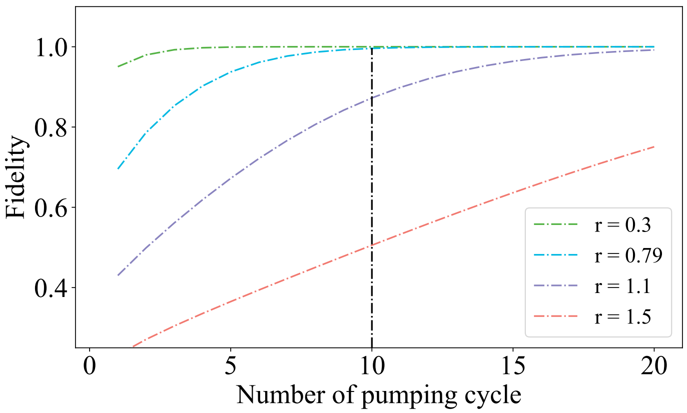

Figure S1: **The relationship between state fidelity and the number of dissipation cycles.** The simulated result of the dissipative process with squeezed parameter $r = 0.1, 0.79, 1.1$ and 1.5. More pumping cycle is needed for larger squeezed parameters.

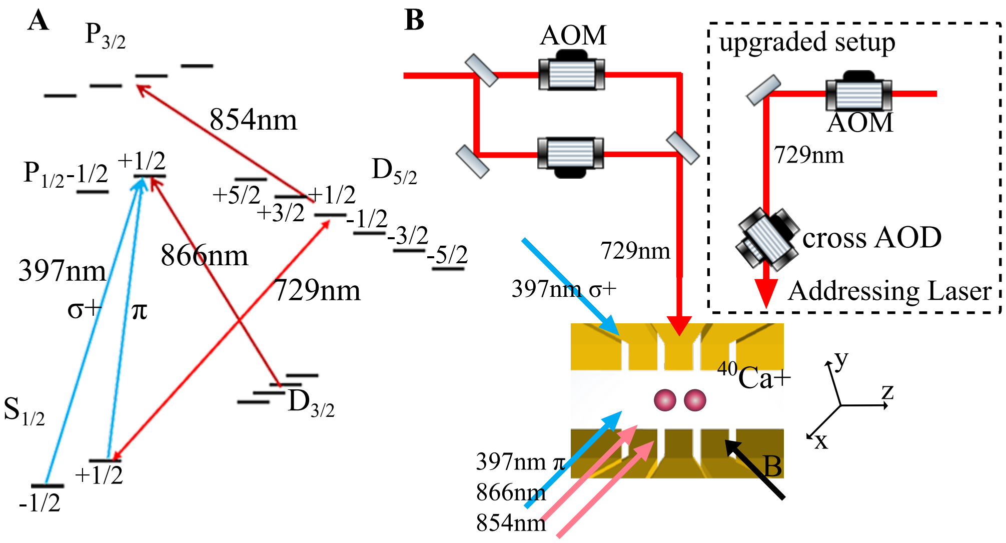

Figure S2: **Experimental setup. A,** the energy level of $^{40}Ca^{+}$. **B,** the optical setup and electronics used in this work. The 729 nm global beam is used for the case with two modes. The addressing beam is used for the case with three and five modes in the upgraded setup.

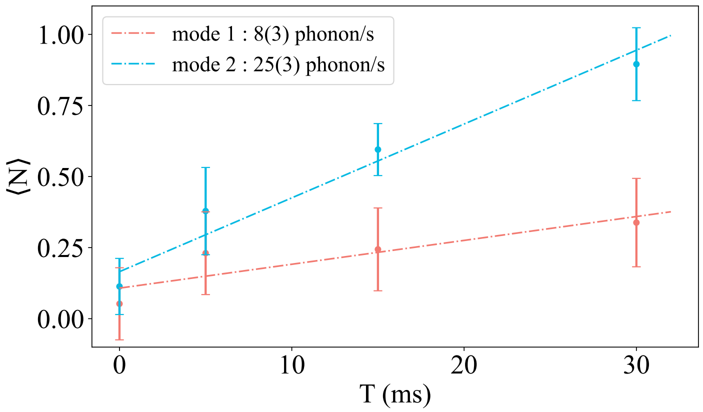

Figure S3: **Heating rate of two motional modes.** The heating rates of a single ion for mode 1 and 2 are 8(3) phonon/s and 25(3)phonon/s respectively, fitted by measuring the average phonon number $\langle N \rangle$ with varied wait time.

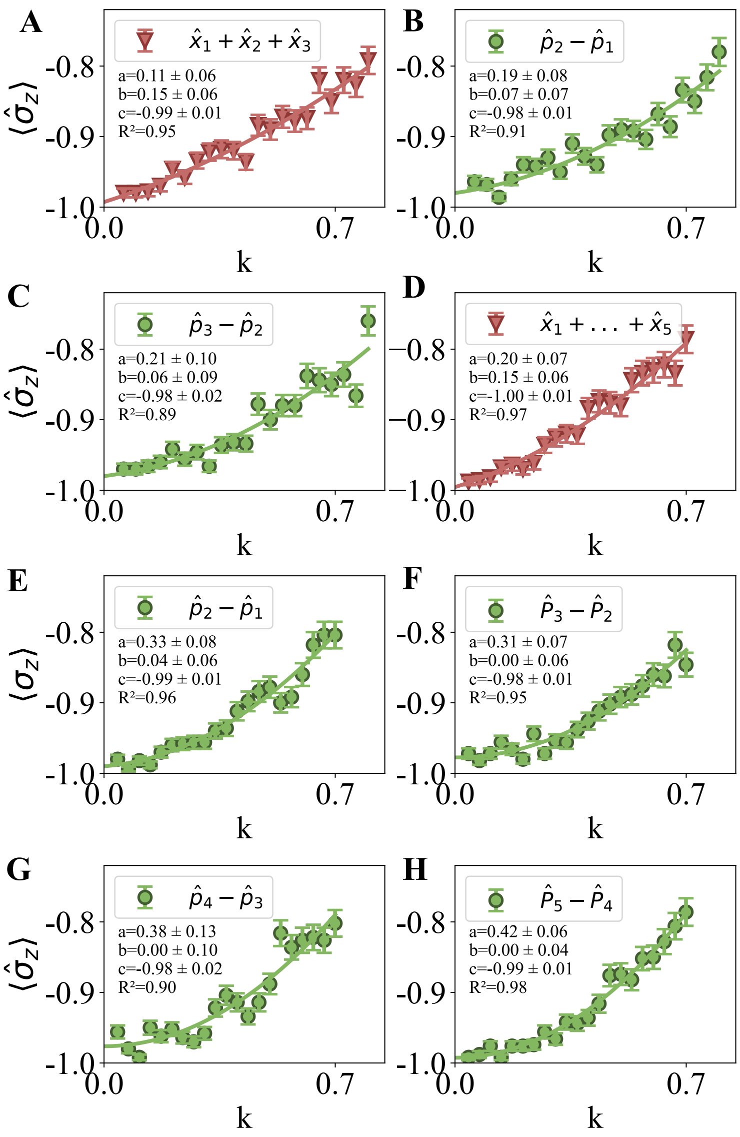

Figure S4: **Spin measurements with varied displacement values k.** The data points depicted in green and red dots correspond to spin-dependent displacements with respect to $\hat{p}_{m+1} - \hat{p}_m$ and $\hat{x}_1 + \hat{x}_2 + ... + \hat{x}_n$. The error bars stand for one standard deviation. We use the fit function $\langle\hat{\sigma}_z\rangle = ak^2 + bk + c$ to obtain the desired variance, whose value is equaled to $2a$, as described in the text. We show the fitted values, corresponding standard errors, and coefficients of determination (R^2). Panel (A)-(C) for the three-mode squeezed state. The fitting results are $\langle(\hat{x}_1+\hat{x}_2+\hat{x}_3)^2\rangle = 0.23\pm0.13$, $\langle(\hat{p}_2-\hat{p}_1)^2\rangle = 0.37\pm0.16$ and $\langle(\hat{p}_3-\hat{p}_2)^2\rangle = 0.41\pm0.19$. Panel (D)-(H) for the five-mode squeezed state. The fitting results are $\langle(\hat{x}_1+\hat{x}_2+\hat{x}_3+\hat{x}_4+\hat{x}_5)^2\rangle = 0.41 \pm 0.15$, $\langle(\hat{p}_2-\hat{p}_1)^2\rangle = 0.66 \pm 0.16$, $\langle(\hat{p}_3-\hat{p}_2)^2\rangle = 0.62 \pm 0.15$, $\langle(\hat{p}_4-\hat{p}_3)^2\rangle = 0.76 \pm 0.25$ and $\langle(\hat{p}_5-\hat{p}_4)^2\rangle = 0.84 \pm 0.12$.

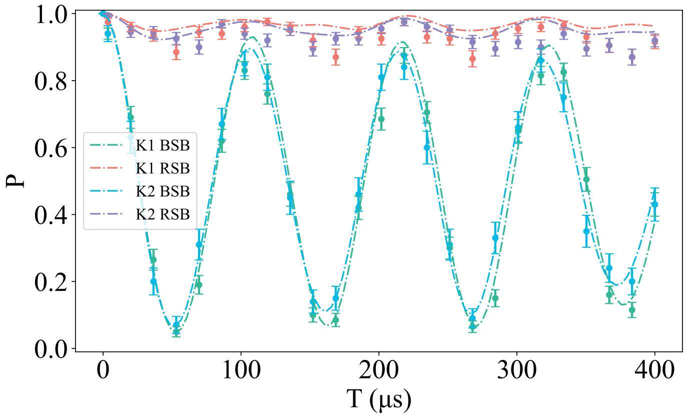

Figure S5: **State analysis in engineered basis.** The experimental data and fitting results using $\hat{H}^{+}_{1,2}$ as probe pulse, labeled with K1, 2 BSB, with varied duration. In contrast, applying $\hat{H}^{-}_{1,2}$ instead provides a minimal change in the spin population, labeled with K1, 2 RSB, indicating the generation of a stabilized state. Each point is obtained from 200 repetitions.

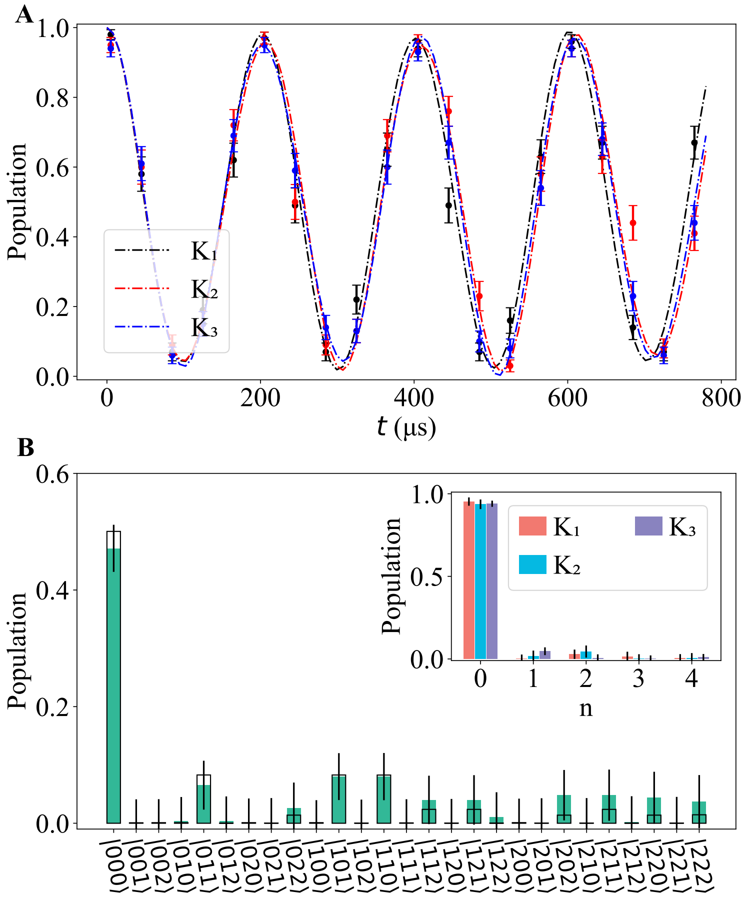

Figure S6: **Experimental results of three-mode squeezed states. A,** Blue sideband transition in the engineered basis. **B,** Population fitting results in Fock basis. The green bars show the experimental results and the black frame shows the population of ideal three-mode squeezed stats. The inset figure shows the population fitting results in an engineered basis.

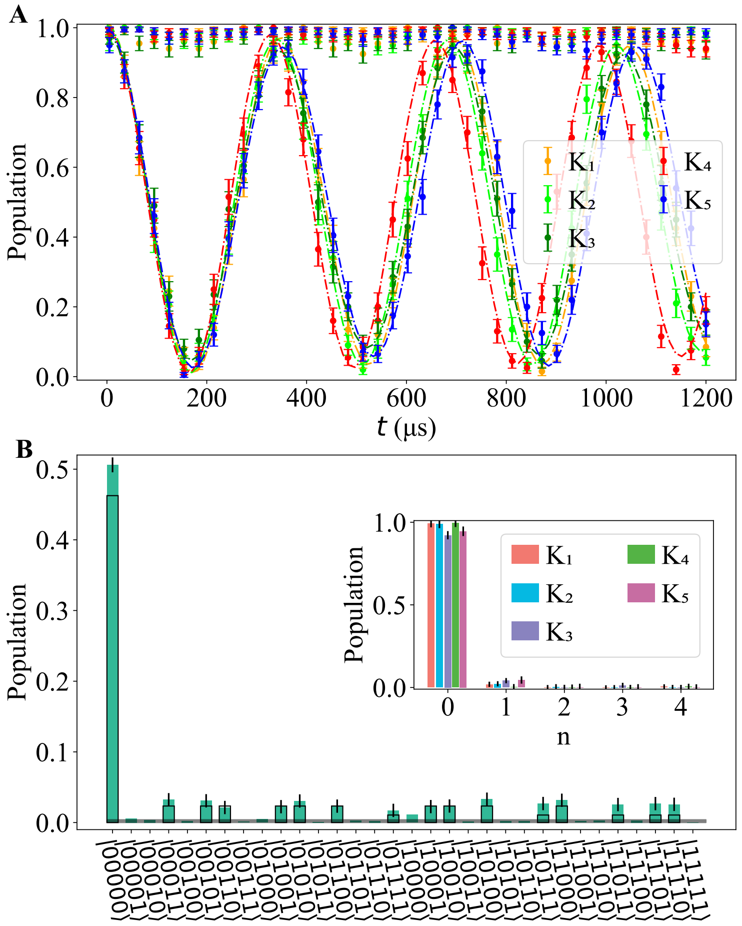

Figure S7: **Experimental results of five-mode squeezed states. A,** Blue sideband transition in the engineered basis. **B,** Population in Fock basis. The green bars show the experimental results and the black frame shows the population of ideal five-mode squeezed states. The shadow indicates the state preparation and measurement (SPAM) error $\sim 0.3\%$ of a single ion. The inset figure shows the population fitting results in an engineered basis.

REFERENCES AND NOTES

1. R. Horodecki, P. Horodecki, M. Horodecki, K. Horodecki, Quantum entanglement. *Rev. Mod. Phys.* **81**, 865–942 (2009).

2. D. M. Greenberger, M. A. Horne, A. Shimony, A. Zeilinger, Bell's theorem without inequalities. *Am. J. Phys.* **58**, 1131–1143 (1990).

3. H. J. Briegel, D. E. Browne, W. Dür, R. Raussendorf, M. V. den Nest, Measurement-based quantum computation. *Nat. Phys.* **5**, 19–26 (2009).

4. H. J. Kimble, The quantum internet. *Nature* **453**, 1023–1030 (2008).

5. I. Buluta, F. Nori, Quantum simulators. *Science* **326**, 108–111 (2009).

6. V. Giovannetti, S. Lloyd, L. Maccone, Advances in quantum metrology. *Nat. Photon.* **5**, 222–229 (2011).

7. S. Cao, B. Wu, F. Chen, M. Gong, Y. Wu, Y. Ye, C. Zha, H. Qian, C. Ying, S. Guo, Q. Zhu, H. L. Huang, Y. Zhao, S. Li, S. Wang, J. Yu, D. Fan, D. Wu, H. Su, H. Deng, H. Rong, Y. Li, K. Zhang, T. H. Chung, F. Liang, J. Lin, Y. Xu, L. Sun, C. Guo, N. Li, Y. H. Huo, C. Z. Peng, C. Y. Lu, X. Yuan, X. Zhu, J.-W. Pan, Generation of genuine entanglement up to 51 superconducting qubits. *Nature* **619**, 738–742 (2023).

8. S. A. Moses, C. H. Baldwin, M. S. Allman, R. Ancona, L. Ascarrunz, C. Barnes, J. Bartolotta, B. Bjork, P. Blanchard, M. Bohn, J. G. Bohnet, N. C. Brown, N. Q. Burdick, W. C. Burton, S. L. Campbell, J. P. Campora, C. Carron, J. Chambers, J. W. Chan, Y. H. Chen, A. Chernoguzov, E. Chertkov, J. Colina, J. P. Curtis, R. Daniel, M. DeCross, D. Deen, C. Delaney, J. M. Dreiling, C. T. Ertsgaard, J. Esposito, B. Estey, M. Fabrikant, C. Figgatt, C. Foltz, M. Foss-Feig, D. Francois, J. P. Gaebler, T. M. Gatterman, C. N. Gilbreth, J. Giles, E. Glynn, A. Hall, A. M. Hankin, A. Hansen, D. Hayes, B. Higashi, I. M. Hoffman, B. Horning, J. J. Hout, R. Jacobs, J. Johansen, L. Jones, J. Karcz, T. Klein, P. Lauria, P. Lee, D. Liefer, S. T. Lu, D. Lucchetti, C. Lytle, A. Malm, M. Matheny, B. Mathewson, K. Mayer, D. B. Miller, M. Mills, B. Neyenhuis, L. Nugent, S. Olson, J. Parks, G. N. Price, Z. Price, M. Pugh, A. Ransford, A. P. Reed, C. Roman, M. Rowe, C. Ryan-Anderson, S. Sanders, J. Sedlacek, P. Shevchuk, P.

Siegfried, T. Skripka, B. Spaun, R. T. Sprenkle, R. P. Stutz, M. Swallows, R. I. Tobey, A. Tran, T. Tran, E. Vogt, C. Volin, J. Walker, A. M. Zolot, J. M. Pino, A race-track trapped-ion quantum processor. *Phys. Rev. X* **13**, 041052 (2023).

9. C. Miller, A. N. Carroll, J. Lin, H. Hirzler, H. Gao, H. Zhou, M. D. Lukin, J. Ye, Two-axis twisting using Floquet-engineered XYZ spin models with polar molecules. *Nature* **633**, 332–337 (2024).

10. X.-Y. Luo, Y. Q. Zou, L.-N. Wu, Q. Liu, M.-F. Han, M. K. Tey, L. You, Deterministic entanglement generation from driving through quantum phase transitions. *Science* **355**, 620–623 (2017).

11. R. McConnell, H. Zhang, J. Hu, S. Ćuk, V. Vuletić, Entanglement with negative Wigner function of almost 3,000 atoms heralded by one photon. *Nature* **519**, 439–442 (2015).

12. P. Thomas, L. Ruscio, O. Morin, G. Rempe, Efficient generation of entangled multiphoton graph states from a single atom. *Nature* **608**, 677–681 (2022).

13. J. F. Poyatos, J. I. Cirac, P. Zoller, Quantum reservoir engineering with laser cooled trapped ions. *Phys. Rev. Lett.* **77**, 4728–4731 (1996).

14. F. Verstraete, M. M. Wolf, J. I. Cirac, Quantum computation and quantum-state engineering driven by dissipation. *Nat. Phys.* **5**, 633–636 (2009).

15. P. M. Harrington, E. J. Mueller, K. W. Murch, Engineered dissipation for quantum information science. *Nat. Rev. Phys.* **4**, 660–671 (2022).

16. G. Morigi, J. Eschner, C. Cormick, Y. Lin, D. Leibfried, D. J. Wineland, Dissipative quantum control of a spin chain. *Phys. Rev. Lett.* **115**, 200502 (2015).

17. J. T. Barreiro, M. Müller, P. Schindler, D. Nigg, T. Monz, M. Chwalla, M. Hennrich, C. F. Roos, P. Zoller, R. Blatt, An open-system quantum simulator with trapped ions. *Nature* **470**, 486–491 (2011).

18. M. Malinowski, C. Zhang, V. Negnevitsky, I. Rojkov, F. Reiter, T. L. Nguyen, M. Stadler, D. Kienzler, K. K. Mehta, J. P. Home, Generation of a maximally entangled state using collective optical pumping. *Phys. Rev. Lett.* **128**, 080503 (2022).

19. Y. Lin, J. P. Gaebler, F. Reiter, T. R. Tan, R. Bowler, A. S. Sørensen, D. Leibfried, D. J. Wineland, Dissipative production of a maximally entangled steady state of two quantum bits. *Nature* **504**, 415–418 (2013).

20. D. C. Cole, S. D. Erickson, G. Zarantonello, K. P. Horn, P. Y. Hou, J. J. Wu, D. H. Slichter, F. Reiter, C. P. Koch, D. Leibfried, Resource-efficient dissipative entanglement of two trapped-ion qubits. *Phys. Rev. Lett.* **128**, 080502 (2022).

21. S. Shankar, M. Hatridge, Z. Leghtas, K. M. Sliwa, A. Narla, U. Vool, S. M. Girvin, L. Frunzio, M. Mirrahimi, M. H. Devoret, Autonomously stabilized entanglement between two superconducting quantum bits. *Nature* **504**, 419–422 (2013).

22. C. Chen, K. Tang, Y. Zhou, K. Y. Yi, X. Zhang, X. Zhang, H. Guo, S. Liu, Y. Chen, T. Yan, D. Yu, Hardware-efficient stabilization of entanglement via engineered dissipation in superconducting circuits. *Phys. Rev. Res.* **7**, L022018 (2025).

23. X. Mi, A. A. Michailidis, S. Shabani, K. C. Miao, P. V. Klimov, J. Lloyd, E. Rosenberg, R. Acharya, I. Aleiner, T. I. Andersen, M. Ansmann, F. Arute, K. Arya, A. Asfaw, J. Atalaya, J. C. Bardin, A. Bengtsson, G. Bortoli, A. Bourassa, J. Bovaird, L. Brill, M. Broughton, B. B. Buckley, D. A. Buell, T. Burger, B. Burkett, N. Bushnell, Z. Chen, B. Chiaro, D. Chik, C. Chou, J. Cogan, R. Collins, P. Conner, W. Courtney, A. L. Crook, B. Curtin, A. G. Dau, D. M. Debroy, A. del Toro Barba, S. Demura, A. di Paolo, I. K. Drozdov, A. Dunsworth, C. Erickson, L. Faoro, E. Farhi, R. Fatemi, V. S. Ferreira, L. F. Burgos, E. Forati, A. G. Fowler, B. Foxen, É. Genois, W. Giang, C. Gidney, D. Gilboa, M. Giustina, R. Gosula, J. A. Gross, S. Habegger, M. C. Hamilton, M. Hansen, M. P. Harrigan, S. D. Harrington, P. Heu, M. R. Hoffmann, S. Hong, T. Huang, A. Huff, W. J. Huggins, L. B. Ioffe, S. V. Isakov, J. Iveland, E. Jeffrey, Z. Jiang, C. Jones, P. Juhas, D. Kafri, K. Kechedzhi, T. Khattar, M. Khezri, M. Kieferová, S. Kim, A. Kitaev, A. R. Klots, A. N. Korotkov, F. Kostritsa, J. M. Kreikebaum, D. Landhuis, P. Laptev, K. M. Lau, L. Laws, J. Lee, K. W. Lee, Y. D. Lensky, B. J. Lester, A. T. Lill, W. Liu, A. Locharla, F. D. Malone, O. Martin, J. R. McClean, M. McEwen, A.

Mieszala, S. Montazeri, A. Morvan, R. Movassagh, W. Mruczkiewicz, M. Neeley, C. Neill, A. Nersisyan, M. Newman, J. H. Ng, A. Nguyen, M. Nguyen, M. Y. Niu, T. E. O'Brien, A. Opremcak, A. Petukhov, R. Potter, L. P. Pryadko, C. Quintana, C. Rocque, N. C. Rubin, N. Saei, D. Sank, K. Sankaragomathi, K. J. Satzinger, H. F. Schurkus, C. Schuster, M. J. Shearn, A. Shorter, N. Shutty, V. Shvarts, J. Skruzny, W. C. Smith, R. Somma, G. Sterling, D. Strain, M. Szalay, A. Torres, G. Vidal, B. Villalonga, C. V. Heidweiller, T. White, B. W. K. Woo, C. Xing, Z. J. Yao, P. Yeh, J. Yoo, G. Young, A. Zalcman, Y. Zhang, N. Zhu, N. Zobrist, H. Neven, R. Babbush, D. Bacon, S. Boixo, J. Hilton, E. Lucero, A. Megrant, J. Kelly, Y. Chen, P. Roushan, V. Smelyanskiy, D. A. Abanin, Stable quantum-correlated many-body states through engineered dissipation. *Science* **383**, 1332–1337 (2024).

24. F. Reiter, D. Reeb, A. S. Sørensen, Scalable dissipative preparation of many-body entanglement. *Phys. Rev. Lett.* **117**, 040501 (2016).

25. M. Raghunandan, F. Wolf, C. Ospelkaus, P. O. Schmidt, H. Weimer, Initialization of quantum simulators by sympathetic cooling. *Sci. Adv.* **6**, eaaw9268 (2020).

26. J. I. Cirac, A. S. Parkins, R. Blatt, P. Zoller, "Dark" squeezed states of the motion of a trapped ion. *Phys. Rev. Lett.* **70**, 556–559 (1993).

27. D. Kienzler, H. Y. Lo, B. Keitch, L. de Clercq, F. Leupold, F. Lindenfelser, M. Marinelli, V. Negnevitsky, J. P. Home, Quantum harmonic oscillator state synthesis by reservoir engineering. *Science* **347**, 53–56 (2015).

28. C. F. Ockeloen-Korppi, E. Damskägg, J. M. Pirkkalainen, M. Asjad, A. A. Clerk, F. Massel, M. J. Woolley, M. A. Sillanpää, Stabilized entanglement of massive mechanical oscillators. *Nature* **556**, 478–482 (2018).

29. S. Barzanjeh, E. S. Redchenko, M. Peruzzo, M. Wulf, D. P. Lewis, G. Arnold, J. M. Fink, Stationary entangled radiation from micromechanical motion. *Nature* **570**, 480–483 (2019).

30. H. Krauter, C. A. Muschik, K. Jensen, W. Wasilewski, J. M. Petersen, J. I. Cirac, E. S. Polzik, Entanglement generated by dissipation and steady state entanglement of two macroscopic objects. *Phys. Rev. Lett.* **107**, 080503 (2011).

31. S. Zippilli, D. Vitali, Dissipative engineering of Gaussian entangled states in harmonic lattices with a single-site squeezed reservoir. *Phys. Rev. Lett.* **126**, 020402 (2021).

32. N. Yazdi, J. J. García-Ripoll, D. Porras, C. Navarrete-Benlloch, Cooling microwave fields into general multimode Gaussian states. *New J. Phys.* **25**, 083052 (2023).

33. N. Yazdi, S. Zippilli, D. Vitali, Generation of stable Gaussian cluster states in optomechanical systems with multifrequency drives. *Quantum Sci. Technol.* **9**, 035001 (2024).

34. J. Han, W. Cai, L. Hu, X. Mu, Y. Ma, Y. Xu, W. Wang, H. Wang, Y. P. Song, C. L. Zou, L. Sun, Experimental simulation of open quantum system dynamics via trotterization. *Phys. Rev. Lett.* **127**, 020504 (2021).

35. X. Ma, W. Rhodes, Multimode squeeze operators and squeezed states. *Phys. Rev. A* **41**, 4625–4631 (1990).

36. S. L. Braunstein, P. van Loock, Quantum information with continuous variables. *Rev. Mod. Phys.* **77**, 513–577 (2005).

37. O. Pfister, Continuous-variable quantum computing in the quantum optical frequency comb. *J. Phys. B: At. Mol. Opt. Phys.* **53**, 012001 (2020).

38. P. van Loock, A. Furusawa, Detecting genuine multipartite continuous-variable entanglement. *Phys. Rev. A* **67**, 052315 (2003).

39. S. Armstrong, J.-F. Morizur, J. Janousek, B. Hage, N. Treps, P. K. Lam, H.-A. Bachor, Programmable multimode quantum networks. *Nat. Commun.* **3**, 1026 (2012).

40. S. Takeda, K. Takase, A. Furusawa, On-demand photonic entanglement synthesizer. *Sci. Adv.* **5**, eaaw4530 (2019).

41. W. Cai, X. Mu, W. Wang, J. Zhou, Y. Ma, X. Pan, Z. Hua, X. Liu, G. Xue, H. Yu, H. Wang, Y. Song, C. L. Zou, L. Sun, Protecting entanglement between logical qubits via quantum error correction. *Nat. Phys.* **20**, 1022–1026 (2024).

42. E. S. Cooper, P. Kunkel, A. Periwal, M. Schleier-Smith, Graph states of atomic ensembles engineered by photon-mediated entanglement. *Nat. Phys.* **20**, 770–775 (2024).

43. C. F. Roos, Controlling the quantum state of trapped ions, thesis, Universität Innsbruck, Austria (2000).

44. C. F. Roos, D. Leibfried, A. Mundt, F. Schmidt-Kaler, J. Eschner, R. Blatt, Experimental demonstration of ground state laser cooling with electromagnetically induced transparency. *Phys. Rev. Lett.* **85**, 5547–5550 (2000).

45. Y. Lin, J. P. Gaebler, T. R. Tan, R. Bowler, J. D. Jost, D. Leibfried, D. J. Wineland, Sympathetic electromagnetically-induced-transparency laser cooling of motional modes in an ion chain. *Phys. Rev. Lett.* **110**, 153002 (2013).

46. D. J. Wineland, C. Monroe, W. M. Itano, D. Leibfried, B. E. King, D. M. Meekhof, Experimental issues in coherent quantum-state manipulation of trapped atomic ions. *J. Res. Natl. Inst. Stand. Technol.* **103**, 259–328 (1998).

47. L.-M. Duan, G. Giedke, J. I. Cirac, P. Zoller, Inseparability criterion for continuous variable systems. *Phys. Rev. Lett.* **84**, 2722 (2000).

48. R. Gerritsma, G. Kirchmair, F. Zähringer, E. Solano, R. Blatt, C. F. Roos, Quantum simulation of the Dirac equation. *Nature* **463**, 68–71 (2010).

49. J. Whitlow, Z. Jia, Y. Wang, C. Fang, J. Kim, K. R. Brown, Quantum simulation of conical intersections using trapped ions. *Nat. Chem.* **15**, 1509–1514 (2023).

50. M. V. Larsen, X. Guo, C. R. Breum, J. S. Neergaard-Nielsen, U. L. Andersen, Deterministic generation of a two-dimensional cluster state. *Science* **366**, 369–372 (2019).

51. W. Asavanant, Y. Shiozawa, S. Yokoyama, B. Charoensombutamon, H. Emura, R. N. Alexander, S. Takeda, J. I. Yoshikawa, N. C. Menicucci, H. Yonezawa, A. Furusawa, Generation of time-domain-multiplexed two-dimensional cluster state. *Science* **366**, 373–376 (2019).

52. N. Shettell, D. Markham, Graph states as a resource for quantum metrology. *Phys. Rev. Lett.* **124**, 110502 (2020).

53. B. K. Malia, Y. Wu, J. Martínez-Rincón, M. A. Kasevich, Distributed quantum sensing with mode-entangled spin-squeezed atomic states. *Nature* **612**, 661–665 (2022).

54. O. Băzăvan, S. Saner, D. J. Webb, E. M. Ainley, P. Drmota, D. P. Nadlinger, G. Araneda, D. M. Lucas, C. J. Ballance, R. Srinivas, Squeezing, trisqueezing, and quadsqueezing in a spin-oscillator system. arXiv:2403.05471 [quant-ph] (2024).

55. I. Rojkov, M. Simoni, E. Zapusek, F. Reiter, J. Home, Stabilization of cat-state manifolds using nonlinear reservoir engineering. arXiv:2407.18087 [quant-ph] (2024).

56. D. Leibfried, R. Blatt, C. Monroe, D. Wineland, Quantum dynamics of single trapped ions. *Rev. Mod. Phys.* **75**, 281–324 (2003).

57. F. Wolf, C. Shi, J. C. Heip, M. Gessner, L. Pezzè, A. Smerzi, M. Schulte, K. Hammerer, P. O. Schmidt, Motional Fock states for quantum-enhanced amplitude and phase measurements with trapped ions. *Nat. Commun.* **10**, 2929 (2019).

58. A. H. Myerson, D. J. Szwer, S. C. Webster, D. T. C. Allcock, M. J. Curtis, G. Imreh, J. A. Sherman, D. N. Stacey, A. M. Steane, D. M. Lucas, High-fidelity readout of trapped-ion qubits. *Phys. Rev. Lett.* **100**, 200502 (2008).

59. C. Weedbrook, S. Pirandola, R. García-Patrón, N. J. Cerf, T. C. Ralph, J. H. Shapiro, S. Lloyd, Gaussian quantum information. *Rev. Mod. Phys.* **84**, 621–669 (2012).

60. W. Chen, Y. Lu, S. Zhang, K. Zhang, G. Huang, M. Qiao, X. Su, J. Zhang, J. N. Zhang, L. Banchi, M. S. Kim, K. Kim, Scalable and programmable phononic network with trapped ions. *Nat. Phys.* **19**, 877–883 (2023).

61. M. Harlander, R. Lechner, M. Brownnutt, R. Blatt, W. Hänsel, Trapped-ion antennae for the transmission of quantum information. *Nature* **471**, 200–203 (2011).

62. M. Valentini, M. W. van Mourik, F. Butt, J. Wahl, M. Dietl, M. Pfeifer, F. Anmasser, Y. Colombe, C. Rössler, P. Holz, R. Blatt, M. Müller, T. Monz, P. Schindler, Demonstration of two-dimensional connectivity for a scalable error-corrected ion-trap quantum processor architecture. arXiv:2406.02406 [quant-ph] (2024).
